# Emotionalität in der COVID-19-Krisenkommunikation von Behörden und unabhängigen Expert*innen auf Twitter: Eine Sentiment-Analyse für das erste Pandemiejahr

**DOI:** 10.1007/s00103-023-03699-z

**Published:** 2023-05-16

**Authors:** Larissa S. Drescher, Jutta Roosen, Katja Aue, Kerstin Dressel, Wiebke Schär, Anne Götz

**Affiliations:** 1 C³ team GbR, Zennerstr. 13, 81379 München, Deutschland; 2 TUM School of Management, Lehrstuhl für Marketing und Konsumforschung, Technische Universität München, Alte Akademie 16, 85354 Freising, Deutschland; 3 Süddeutsches Institut für empirische Sozialforschung e. V., Schwanthalerstr. 91, 80336 München, Deutschland

**Keywords:** Öffentlichkeitsarbeit, SARS-CoV-2, Behördenkommunikation, Expertenkommunikation, Social Media Analytics, Sentiment, Public Relations, SARS-CoV-2, Authorities communication, Experts communications, Social Media Analytics, Sentiments

## Abstract

**Hintergrund:**

Zu Beginn der COVID‑19-Pandemie herrschte in Deutschland große Unsicherheit in der Bevölkerung und bei den für die Krisenkommunikation Verantwortlichen. Ein wesentlicher Teil der Kommunikation von Expert*innen und zuständigen Behörden fand in den sozialen Medien statt, insbesondere auf der Plattform Twitter. Die dort mit der Krisenkommunikation transportierten positiven, negativen und neutralen Sentiments (Emotionen) sind für Deutschland bisher nicht vergleichend untersucht worden.

**Ziel der Arbeit:**

Die Sentiments in Twitter-Meldungen von verschiedenen (Gesundheits‑)Behörden und unabhängigen Expert*innen zu COVID‑19 sollen für das erste Pandemiejahr (01.01.2020–15.01.2021) ausgewertet werden, um eine Wissensgrundlage für die Verbesserung zukünftiger Krisenkommunikation zu schaffen.

**Material und Methoden:**

Von *n* = 39 Twitter-Akteur*innen (21 Behörden und 18 Expert*innen) flossen *n* = 8251 Tweets in die Auswertung ein. Diese erfolgte mit dem sog. Lexikonansatz, einer Methode der Social-Media-Analyse. Es wurden deskriptive Statistiken berechnet u. a. zur Bestimmung der durchschnittlichen Polarität der Sentiments und der Häufigkeiten positiv und negativ besetzter Wörter in 3 Phasen der Pandemie.

**Ergebnisse und Diskussion:**

Die Entwicklung der Emotionalität in COVID‑19-Tweets und der Anzahl von Neuinfektionen in Deutschland verlaufen in etwa parallel. Die Analyse zeigt, dass die Polarität der Sentiments bei beiden Akteursgruppen im Durchschnitt negativ ist. Expert*innen twittern im Untersuchungszeitraum deutlich negativer über COVID‑19 als Behörden. Behörden kommunizieren in der zweiten Phase nahe der Neutralitätslinie, also weder ausgeprägt positiv noch negativ.

## Abstract (en)

**Background:**

At the beginning of the COVID‑19 pandemic in Germany, there was great uncertainty among the population and among those responsible for crisis communication. A substantial part of the communication from experts and the responsible authorities took place on social media, especially on Twitter. The positive, negative, and neutral sentiments (emotions) conveyed there during crisis communication have not yet been comparatively studied for Germany.

**Study aim:**

Sentiments in Twitter messages from various (health) authorities and independent experts on COVID‑19 will be evaluated for the first pandemic year (1 January 2020 to 15 January 2021) to provide a knowledge base for improving future crisis communication.

**Material and methods:**

From *n* = 39 Twitter actors (21 authorities and 18 experts), *n* = 8251 tweets were included in the analysis. The sentiment analysis was done using the so-called lexicon approach, a method within the social media analytics framework to detect sentiments. Descriptive statistics were calculated to determine, among other things, the average polarity of sentiments and the frequencies of positive and negative words in the three phases of the pandemic.

**Results and discussion:**

The development of emotionality in COVID‑19 tweets and the number of new infections in Germany run roughly parallel. The analysis shows that the polarity of sentiments is negative on average for both groups of actors. Experts tweet significantly more negatively about COVID‑19 than authorities during the study period. Authorities communicate close to the neutrality line in the second phase, that is, neither distinctly positive nor negative.

## Hintergrund

Die für Krisen charakteristische Unsicherheit und eine sich ständig und schnell ändernde Sachlage machen die Kommunikation neuer wissenschaftlicher Erkenntnisse durch (Gesundheits‑)Behörden an die Bevölkerung dringlich. Dies wurde auch 2020 beim Aufkommen des Coronavirus SARS-CoV‑2 in Deutschland deutlich. Insbesondere Social-Media-Plattformen wie Twitter spielen bei der Informationsverbreitung von Behörden und der Informationssuche der Bevölkerung eine große Rolle [1–3]. Soziale Medien sind eine wichtige Informationsquelle und Bestandteil der Krisenkommunikation, allerdings nur für einen Teil der Bevölkerung [4]. Zum Zeitpunkt der Erhebung wurde Twitter von weniger als 10 % der deutschen Bevölkerung genutzt [5]. Eine Twitter-Analyse ist entsprechend zwar nicht repräsentativ für die gesamte Bevölkerung, es befinden sich aber besonders aktive und interessierte Akteur*innen mit Sendungsbewusstsein und politischem Interesse auf dieser Plattform [6, 7]. Twitter ist ein immer wichtiger werdendes Medium der Krisenkommunikation [8].

Neben Behörden, also staatlichen und kommunalen Institutionen, die im öffentlichen Auftrag informieren und kommunizieren, sind es auf Social-Media-Plattformen vor allem Wissenschaftler*innen, Wissenschaftsjournalist*innen und Politiker*innen, die die öffentliche Diskussion rund um COVID‑19 in ihrer Funktion als unabhängig und eigenverantwortlich kommunizierende Expert*innen dominieren [8]. Auch finden die öffentlich geführten Diskurse zunehmend abseits traditioneller Medien statt und Politik und Zivilgesellschaft können mittels sozialer Medien in direkten Austausch treten. Dadurch verliert der klassische Journalismus seine alleinige Gatekeeper-Funktion [9].

Eine wissenschaftliche Definition des Begriffs „Expert*in“ vor dem Hintergrund der Social-Media-Kommunikation von Wissenschaftler*innen liegt bisher nicht vor. Bogner et al. [10] definieren den Begriff Experte folgendermaßen: „Experten lassen sich als Personen verstehen, die sich – ausgehend von einem spezifischen Praxis- oder Erfahrungswissen, das sich auf einen klar begrenzbaren Problemkreis bezieht – die Möglichkeit geschaffen haben, mit ihren Deutungen das konkrete Handlungsfeld sinnhaft und handlungsleitend für andere zu strukturieren.“ In diesem Beitrag werden unter unabhängigen Expert*innen daher solche Einzelpersonen verstanden, die aufgrund ihrer Erfahrung und Reputation zum Coronavirus auf Twitter sichtbar sind. Wir bezeichnen sie als unabhängig, da sie mit einem privaten, öffentlichen Twitter-Account und nicht im Namen ihrer Institution kommunizieren.

Sowohl Behörden als auch unabhängige Expert*innen haben Informationen zur COVID‑19-Pandemie ungefiltert und direkt mit der Twitter-Community geteilt. Im Rahmen der Pandemie in Deutschland haben Wissenschaftler*innen auch interne Debatten in einem starken Ausmaß öffentlich und in Echtzeit ausgetragen. An dieser Stelle sei darauf hingewiesen, dass auch in Behörden Expert*innen arbeiten, hier aber eine Unterscheidung vorgenommen wird zwischen Behörden, die als sichtbare Organisationseinheit auf Twitter kommunizieren, und Expert*innen, die zwar Teil einer Behörde sein können, aber eigenständig verantwortlich mit persönlichem Account als Expert*in auf Twitter kommunizieren, auch wenn sie ihre institutionelle Zugehörigkeit offenlegen.

Bereits vor der COVID‑19-Pandemie hat sich Twitter als ein sehr erfolgreiches Tool zur Krisenkommunikation erwiesen: Gesundheitliche Krisensituationen, Naturkatastrophen, Terroranschläge oder soziale Ereignisse sind auf Twitter verfolgbar. Diverse Studien zur Analyse von Krisenkommunikation liegen vor [11–14], so auch verschiedene internationale Twitter-Untersuchungen zur COVID‑19-Krisenkommunikation [2, 4, 15, 16].

In einer vorangegangenen Studie haben die Autor*innen dieses Beitrags mittels quantitativer Inhaltsanalyse untersucht, welche Inhalte die COVID‑19-Tweets von Behörden und unabhängigen Expert*innen thematisieren und wovon deren Verbreitung mittels Retweeten (erneutes Posten) oder Liken (Klick auf „Gefällt mir“) abhängt [17]. Dabei zeigte sich, dass die Krisenkommunikation von unabhängigen Expert*innen bezogen auf die Verbreitung auf Twitter deutlich erfolgreicher ist als die der Behörden: Die Tweets der Expert*innen wurden im ersten Pandemiejahr (2020) 7‑mal häufiger retweetet und 13,9-mal häufiger gelikt als die der Behörden. Es wurde gezeigt, dass der signifikant größere Erfolg der Expert*innen mit der Verwendung bestimmter struktureller Merkmale in Tweets assoziiert ist, wie z. B. mit der Nutzung von Hashtags. So geht die geringere Verwendung von Hashtags in Expert*innen-Tweets einher mit einer größeren Reichweite der COVID‑19-Tweets. Auch andere Untersuchungen kommen zu dem Schluss, dass Behördenposts auf Twitter zu den unbeliebtesten Nachrichten gehören und eine deutlich geringere Reichweite haben [18]. Expert*innen – und hier insbesondere Virolog*innen – gehören dagegen zu den beliebtesten Twitterern [19].

Zur Aufdeckung der Reichweite von Tweets werden quantitative Inhaltsanalysen mit qualitativen Analysen kombiniert [18]. Die in der vorliegenden Arbeit vorgenommene Sentiment-Analyse stellt eine Erweiterung der quantitativen Vorstudie [17] dar, um die Frage zu beantworten, ob der größere Erfolg von Expert*innen-Tweets zu COVID‑19 in Deutschland qualitativ in den Sentiments der Tweets sichtbar wird. Daher gehen wir in einer deskriptiven Analyse der Frage nach, ob Unterschiede in den Sentiments zwischen beiden Akteursgruppen bestehen.

Die sogenannte Sentiment-Analyse ist dem Bereich des Natural Language Processing (NLP)^1^ zuzuordnen und gehört derzeit zu den beliebtesten Analysemethoden für die Auswertung von Social-Media-Daten [20]. Sentiments bzw. Emotionen im Zusammengang mit Twitter-Nachrichten zu COVID‑19 wurden bereits mehrfach untersucht [21–26]. Bei Sentiment-Analysen wird untersucht, ob Personen sich eher mit positiv oder negativ besetzten Wörtern über einen Sachverhalt auf Social-Media-Plattformen äußern (Aufdeckung von Polaritäten). Neben Studien, die Methoden des maschinellen Lernens nutzen [27], ist eine Reihe von COVID‑19-Studien basierend auf dem Lexikonansatz veröffentlicht worden, bei dem definierte Wortlisten genutzt werden [28, 29].

Eine chinesische Sentiment-Analyse zeigt, dass Angst in allen COVID‑19-Tweets dominierte [25]. Eine US-amerikanische Analyse findet, dass negativ konnotierte Sentiments bezüglich COVID‑19 im Verlauf der Pandemie zunahmen und Angst die häufigste Emotion in Twitter-Nachrichten war [26]. Eine Übersicht über die Entwicklung globaler Sentiments der COVID‑19-Pandemie liefern auch Lwin et al. [23] über eine Inhaltsanalyse englischsprachiger Tweets unter Berücksichtigung der Emotionen Angst, Wut, Freude und Traurigkeit. Die Sentiments zu COVID‑19 haben sich laut der Studie innerhalb weniger Wochen stark verändert. Während in der ersten Phase der Pandemie Angst die vorherrschende Emotion war, wurden im Zeitverlauf immer weniger mit Angst besetzte Wörter in Tweets genutzt. Die Emotion Wut hat dagegen zugenommen [23]. Die Studien zeigen insgesamt, dass negative Emotionen in den Twitter-Nachrichten während der Pandemie dominierten.

In der vorliegenden Arbeit wird die Polarität von Emotionen (positiv, negativ, neutral) untersucht, die den von Behörden und unabhängigen Expert*innen verwendeten Wörtern in COVID‑19 Tweets zugeschrieben werden kann. Die Polaritäten der Tweets werden für das erste Pandemiejahr (vom 01.01.2020 bis zum 15.01.2021) berechnet und zwischen Behörden und unabhängigen Expert*innen verglichen. Bei dieser Arbeit handelt es sich um eine Teilstudie der vom Bundesamt für Strahlenschutz (BfS) beauftragten Gesamtstudie „Eine vergleichende Evaluation der Online-Krisenkommunikation von Behörden und unabhängigen Expert*innen im Zuge der COVID‑19 Pandemie als Grundlage für die Verbesserung der BfS-Krisenkommunikation – Los 2“ [30].

Das Ziel der Studie ist die Beschreibung der Sentiments im Zeitablauf der Pandemie, um daraus Erkenntnisse zur Optimierung der behördlichen Krisenkommunikation für künftige (Krisen‑)Ereignisse in Deutschland zu gewinnen. Diese Erkenntnisse sollen die Ergebnisse der vorangegangenen quantitativen Inhaltsanalyse [17] ergänzen, um die Frage zu beantworten, wodurch der größere Erfolg der Expert*innen bei der Verbreitung von Informationen zu COVID‑19 begründet ist.

## Methoden

Im Folgenden wird der in der Studie verwendete Methoden-Mix vorgestellt. Zunächst wird die Akteur*innen-Auswahl von Behörden und Expert*innen erläutert. Es schließt sich eine Darstellung der Twitter-Datengewinnung und der Datenbereinigung sowie der Methoden zur Datenauswertung mittels Sentiment-Analyse an.

### Auswahl der Akteur*innen

Die Auswahl der Twitter-Accounts beider Gruppen war größtenteils extern durch den Auftraggeber der Gesamtstudie (BfS) vorgegeben. Es handelte sich um Behörden und unabhängige Expert*innen, die für den Auftraggeber von Interesse sind. Wie in der Einleitung beschrieben, wurden unter dem Begriff „Behörden“ in diesem Beitrag staatliche und kommunale Institutionen subsummiert, die im öffentlichen Auftrag auf Twitter informieren und kommunizieren. Als Teil des Behördennetzwerkes wurden hier auch wissenschaftliche Einrichtungen erfasst, die nicht als Einzelpersonen twittern, wie z. B. Institute der Helmholtz-Gemeinschaft, die Leopoldina oder die Wissenschaftspressekonferenz. Dieser Gruppe gehören auch Behörden mit hoheitlichen Aufgaben an, wie das Robert Koch-Institut (RKI).

Bei den ausgewählten Expert*innen handelte es sich um Einzelpersonen, die persönlich und damit eigenverantwortlich auf Twitter kommunizieren.^2^ Der Definition von Bogner et al. [10] folgend, wurden die Accounts der unabhängigen Expert*innen basierend auf der *wahrgenommenen* Expertise und einer hohen Reichweite derselben in Abstimmung mit dem Auftraggeber festgelegt. Virolog*innen und Epidemiolog*innen wurden eingeschlossen, aber auch inhaltlich befasste Politiker*innen und Wissenschaftsjournalist*innen. Nicht eingeschlossen wurden Accounts, die sich auf soziale oder wirtschaftliche Folgen der Pandemie bezogen.

### Twitter-Datengewinnung und Bereinigung

Der Abzug der Twitter-Daten aller 39 Akteur*innen fand über eine Programmierschnittstelle (Application Programming Interface, API) statt (Twitter-Developer-Zugang der Autor*innen). Der Datenabzug und die Datenbearbeitung erfolgten über die Software Posit/RStudio Version 1.31093 (Posit Software, PBC, Boston, USA) für Windows und zugehörige Code-Packages sowie Excel für Microsoft 365 (Microsoft Corporation, Redmond, USA).

Twitter ist eine öffentliche Social-Media-Plattform. Für die Extrahierung und Auswertung von Twitter-Daten beschreibt die „Soziale Medien Richtlinie“, herausgegeben von Verbänden der Markt- und Sozialforschung in Deutschland [31], eine Vorgehensweise, der hier gefolgt wurde: „In offenen Sozialen Medien bzw. den entsprechenden Bereichen dürfen die personenbezogenen Daten der Teilnehmer grundsätzlich ohne entsprechende explizite Einwilligung auf der Grundlage der gesetzlichen Erlaubnisnorm auch für Zwecke der Markt- und Sozialforschung verarbeitet und genutzt werden“ [31].

Twitter-Nutzer*innen sollte bekannt sein, dass Twitter eine öffentliche Plattform ist und die Daten öffentlich sind und für wissenschaftliche Zwecke verwendet werden können. Das Unternehmen Twitter selbst weist in den Allgemeinen Geschäftsbedingungen die Nutzer*innen darauf hin [32]. Twitter kann auch für private Nachrichten genutzt werden. Diese privaten Nachrichten waren nicht Gegenstand dieser Teilstudie. Beim Anlegen eines Twitter-Accounts muss den Nutzungsbedingungen zugestimmt werden. Diese sagen weiter aus, dass die von den Nutzer*innen erstellten Tweet-Inhalte in deren eigener Verantwortung liegen und von Twitter anderen Unternehmen und Einzelpersonen zur Verfügung gestellt und dort auch weiterverarbeitet werden können [32]. Greifen Wissenschaftler*innen auf Twitter-Daten zu, so arbeiten sie genau genommen mit öffentlich bereitgestellten Daten.

Der Datenabzug der Tweets wurde am Stichtag 15.01.2021 vollzogen. Für alle Akteur*innen war rein technisch durch Twitter Inc. ein Abzug der letzten 3200 Tweets über ihren Twitter-Nutzernamen/Handle möglich. Das ergab einen Grunddatensatz von *n* = 81.455 Tweets, die in Abhängigkeit von der Häufigkeit des Twitterns zeitlich unterschiedlich weit in die Vergangenheit reichten. Im ersten Schritt wurde der Datensatz auf den für die Analyse festgelegten Zeitrahmen reduziert. Für die Sentiment-Analyse wurde der Zeitraum vom 01.01.2020 bis zum 15.01.2021 betrachtet. Zusätzlich wurde dieser in 3 Phasen aufgeteilt, um die Entwicklung der Sentiments im ersten Pandemiejahr vergleichen zu können. Die Phasen orientieren sich an zentralen Ereignissen:^3^

- Phase 1: 01.01.2020–22.03.2020 (Beginn der Pandemie in Deutschland mit dem ersten Lockdown),
- Phase 2: 23.03.2020–28.10.2020 (Ende des ersten Lockdowns bis Beginn der zweiten Welle),
- Phase 3: 29.10.2020–15.01.2021 (zweite Welle und Aufkommen der Diskussionen um Entwicklung und Verfügbarkeit der Impfstoffe um den Jahreswechsel 2020/2021).

Der nächste Datenbearbeitungsschritt war die Identifizierung von Tweets mit COVID‑19-Bezug, die über das Filtern basierend auf Schlüsselwörtern erfolgte. Das Vorgehen zum Herausfiltern der COVID‑19-Tweets ist detailliert in [17] beschrieben. Die Filterung ergab *n* = 35.645 COVID‑19-Tweets. Für genaue Ergebnisse der Sentiment-Analyse ist ein Fokus auf die von den Akteur*innen originär verfassten Tweets notwendig. Daher fand hierfür eine Bereinigung um Tweets statt, die Quotes (Zitate), Retweets (Weiterleitungen) oder Replies (Antworten) darstellen. Der Datensatz wurde zudem auf deutsche Tweets beschränkt. Damit verblieben *n* = 8251 originär von den Akteur*innen auf Deutsch verfasste COVID‑19-Tweets für die Sentiment-Analyse. Daraus geht hervor, dass nur 23 % der COVID‑19-Tweets des Samples originär verfasst sind. Nur Wörter flossen in die Analysen ein. Eine Untersuchung anderer Stilelemente wie die Verwendung von Bildern oder Memes war nicht Teil dieser Untersuchung, d. h., es wurde nur der reine Text der Tweets untersucht.

### Datenauswertung

In diesem Kapitel wird der Methoden-Mix bei der Datenauswertung beschrieben. Grundlage ist das Social Media Analytics Framework, von dem die Sentiment-Analyse ein Teil ist.

Das Social Media Analytics Framework [35] wird seit den 2010er-Jahren zur Untersuchung von Social-Media-Daten angewandt. Social Media Analytics gilt als vergleichsweise neuer Methoden-Mix [36, 37]. Social Media Analytics „… befasst sich mit der Entwicklung und Evaluierung von Informatikwerkzeugen und Frameworks zur Sammlung, Überwachung, Analyse, Zusammenfassung und Visualisierung von Social-Media-Daten, in der Regel auf der Grundlage spezifischer Anforderungen einer Zielanwendung“ [36]. Es handelt sich um multidisziplinäre Methoden, in denen Beiträge aus der Informatik, Statistik, Netzwerkanalyse und Linguistik kombiniert werden.

Im Folgenden wird die Sentiment-Analyse von Tweets beschrieben. Es gibt verschiedene Ansätze der Auswertung von Sentiments [37]. Zu den methodischen Ansätzen im Bereich der Sentiment-Analyse gehören technisch komplexe Methoden wie maschinelles Lernen („machine learning-based models“). Dabei werden Algorithmen konzipiert, die direkt aus (Twitter‑)Daten Informationen ziehen und dadurch stetig dazulernen. Maschinelles Lernen ist dem Bereich Big Data zuzuordnen und wird auf verschiedenen Gebieten, z. B. in der Analyse von Finanzdaten in der Pandemie, eingesetzt [38]. Eine weitere Möglichkeit ist der in diesem Beitrag genutzte „Lexikonansatz“, der als einfache und zielführende Methode zur Auswertung von Sentiments speziell im Rahmen von COVID‑19- und Twitter-Daten beschrieben wird [29].

In diesem Beitrag wird eine vereinfachte Definition von „Sentiment“ als „Empfindung“ bzw. „Gefühl“ genutzt. Eine „Sentiment-Analyse“ ist die computergestützte Analyse von in Texten ausgedrückten Meinungen, Gefühlen und Emotionen [39]. Das bedeutet, dass es sich bei Sentiments, Gefühlen und Emotionen nicht um dieselben, aber ähnliche Konzepte handelt. Weiter definiert Liu [39] in dem Zusammenhang, dass eine „Meinung“ zu einem Thema also eine positive oder negative Bewertung durch einen Meinungsträger ist. Das heißt, auch Liu spricht von negativen und positiven Emotionen. Emotionen werden verstanden als „subjektive Gefühle und Gedanken“ [39].

Bei einer Auseinandersetzung mit subjektiven Gefühlen, Emotionen oder Meinungen ist es wichtig, für deren Beschreibung zwischen inneren Zuständen und sprachlichen Ausdrücken zu unterscheiden. Die verschiedenen Arten von Gefühlen können auf mannigfaltige Weise sprachlich ausgedrückt werden. Es gibt so auch eine Vielzahl an Meinungsäußerungen, die positive oder negative Emotionen beschreiben [39]. Vor diesem Hintergrund setzen wir Emotionen und Polaritäten (vereinfacht) gleich, weil dies eine Möglichkeit darstellt, die inneren Zustände hinter den in der Sprache ausgedrückten Emotionen zu operationalisieren. Andere Sentiment-Studien nutzen ebenfalls diese vereinfachte Gleichsetzung von Sentiments und Polaritäten [40].

Die Sentiment-Analyse hat eine lange Tradition in der Aufdeckung von Polaritäten, wobei die Analyse der zugrunde liegenden Emotionen lange Zeit schwierig war – und für den deutschsprachigen Raum bis heute ist. Für den englischsprachigen Raum gibt es Emotionslexika [41], in denen z. B. 8 verschiedene emotionale Zustände unterschieden werden. Den Begriffen werden daneben auch positive oder negative Konnotation zugeordnet. Für den deutschsprachigen Raum gibt es nur 2 Sentiment-Lexika: das in diesem Beitrag verwendete SentiWS der Universität Leipzig [42] und ein Lexikon speziell für politische Texte [43]. Auch in diesem Lexikon werden positive und negative Sentiments unterschieden.

Die Auswertung der Emotionen, die vom Text transportiert werden, erfolgte in der vorliegenden Studie basierend auf einer definierten Wortliste (Lexikon), in der die emotionale Bedeutung von deutschen Wörtern als Polarität festgehalten und quantifiziert ist (Positivität, Neutralität, Negativität). Je stärker die zugeordnete Emotion ist, umso positiver/negativer fällt dieser Wert aus.

Für die Auswertung wurde der Text der *n* = 8251 Tweets beider Akteursgruppen in einzelne Wörter aufgeteilt [38] und einer Einzelwortanalyse unterzogen. Für die Berechnung der Sentiments wurde Version 2.0 von SentiWS, einem öffentlich verfügbaren deutschen Lexikon für Sentiment-Analysen von Remus et al. verwendet [42]. Die neueste Version enthält Wörter mit positiver (*n* = 1650) und negativer (*n* = 1800) Polarität, die in einem Intervall von −1 und +1 liegen [42].

Die Sentiment-Werte der Einzelwörter sind zu Sentiments je Tweet addiert worden. Um Trends in den Sentiments von Tweets zu bestimmen, wird typischerweise die durchschnittliche Polarität der Sentiments durch eine Mittelung der Polaritätswerte der Tweets berechnet und im Zeitverlauf verglichen [29, 44]. Entsprechend war das weitere methodische Vorgehen, für alle Tweets der jeweiligen Gruppe die durchschnittliche Polarität der Sentiments zu berechnen. Daneben wurden häufig vorkommende Wörter mit positiver und negativer Konnotation auch einzeln betrachtet.

## Ergebnisse

Eine Übersicht über die 21 Behörden, deren COVID‑19-Tweets untersucht wurden, befindet sich in Tab. 1. Zusätzlich wurden die Tweets von 18 Expert*innen^4^ untersucht. Insgesamt haben diese 39 Akteur*innen vom 01.01.2020 bis zum 15.01.2021 *n* = 8251 originäre COVID‑19-Tweets veröffentlicht.

**Table 1:** 

Behörden (inkl. Einrichtungen im Behördennetzwerk)	Twitter-Nutzername
Bundesamt für Bevölkerungsschutz und Katastrophenhilfe (BBK)	@BBK_Bund
Bundesinstitut für Risikobewertung (BfR)	@BfRde
Bundesministerium für Gesundheit (BMG)	@BMG_Bund
Landesregierung Baden-Württemberg	@RegierungBW
Bundeszentrale für gesundheitliche Aufklärung (BZgA)	@bzga_de
Charité – Universitätsmedizin Berlin	@ChariteBerlin
Hamburger Senat	@Senat_Hamburg
Helmholtz-Gemeinschaft	@helmholtz_de
Helmholtz-Zentrum für Infektionsforschung (HZI)	@Helmholtz_HZI
Landesamt für Gesundheit und Soziales in Berlin (LAGeSo)	@LaGeSo_Berlin
Leopoldina (Nationale Akademie der Wissenschaften Leopoldina)	@Leopldina
LSA (Landesregierung Sachsen-Anhalt)	@sachsenanhalt
Max-Planck-Gesellschaft	@maxplanckpress
Max-Planck-Institut für Infektionsbiologie (MPIIB)	@mpiib_berlin
Stadt München	@StadtMuenchen
Niedersächsische Landesregierung	@NdsLandesReg
Paul-Ehrlich-Institut (PEI)	@PEI_Germany
Robert Koch-Institut (RKI)	@rki_de
Landesregierung Rheinland-Pfalz	@rlpNews
Weltgesundheitsorganisation, Regionalbüro Europa	@WHO_Europe_de
Wissenschafts-Pressekonferenz e. V.	@wpk_daily

### Polarität der Sentiments von Behörden und unabhängigen Expert*innen im Vergleich

Über das erste Pandemiejahr ergibt die Auswertung der Sentiments in den COVID‑19-Tweets für beide Akteursgruppen im Durchschnitt einen negativen Polaritätswert. Dabei liegt der durchschnittliche Wert der Behörden-Tweets bei −0,008, der der Expert*innen-Tweets bei −0,087.^5^ Das verdeutlicht, dass das Textkorpus beider Gruppen insgesamt betrachtet negativ konnotiert ist. Die Tatsache, dass die Werte nah an der Null liegen, ist nicht überraschend, da die Autoren des SentiWS-Lexikons schon darauf hinweisen, dass es nur wenige Wörter mit starken Sentiments gibt (Wörter mit hohen Polaritätswerten, z. B. „Freude“ mit 0,6502 oder „schädlich“ mit −0,9269), während die meisten Wörter geringe oder sehr geringe Werte aufweisen. Das wichtigste Ergebnis ist hier, dass die Polarität der Sentiments im Durchschnitt negativ ist.

**Figure 1:**
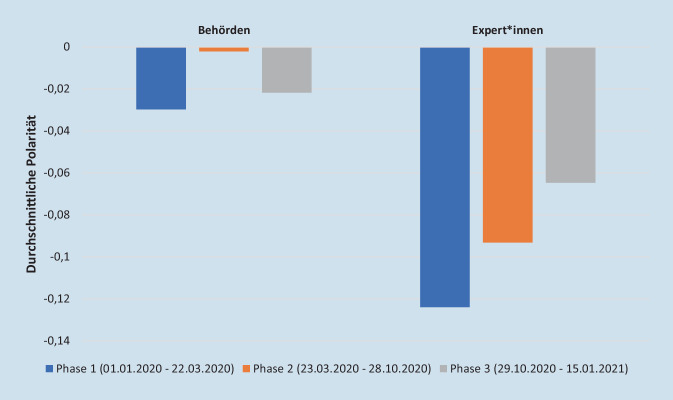

Abb. 1 zeigt die Entwicklung der durchschnittlichen Polarität der Sentiments aufgeteilt in den 3 Phasen der Untersuchung.

In Phase 1 zeigen die COVID‑19-Tweets bei beiden Akteursgruppen die größten Negativwerte. Auffällig ist, dass die Expert*innen-Tweets in allen 3 Phasen deutlich negativer konnotiert sind als die der Behörden. Zwar nimmt die negative Polarität bei den Expert*innen-Tweets im Zeitverlauf ab (sie wird also weniger negativ), allerdings haben deren Tweets in Phase 3 noch immer stärkere Negativwerte als die der Behörden im gesamten Untersuchungszeitraum. Bei den Expert*innen sind die Sentiments auch in der zweiten Phase, inklusive des Sommerplateaus, deutlich negativ.

Die Behörden-Tweets sind weniger negativ konnotiert im Vergleich zu den Expert*innen und zudem zeigt sich, dass die Sentiments in Phase 2 nahezu neutral sind. In diesen Zeitraum fällt auch der Sommer 2020 und das Abflauen der ersten Pandemiewelle. In Phase 3 werden die Behörden-Tweets wieder negativer.

Die Ergebnisse zeigen also, dass die Expert*innen deutlich emotionaler zu COVID‑19 kommunizieren als die Behörden.

### Häufigkeit positiv und negativ besetzter Wörter in den 3 Pandemiephasen

**Figure 2:**
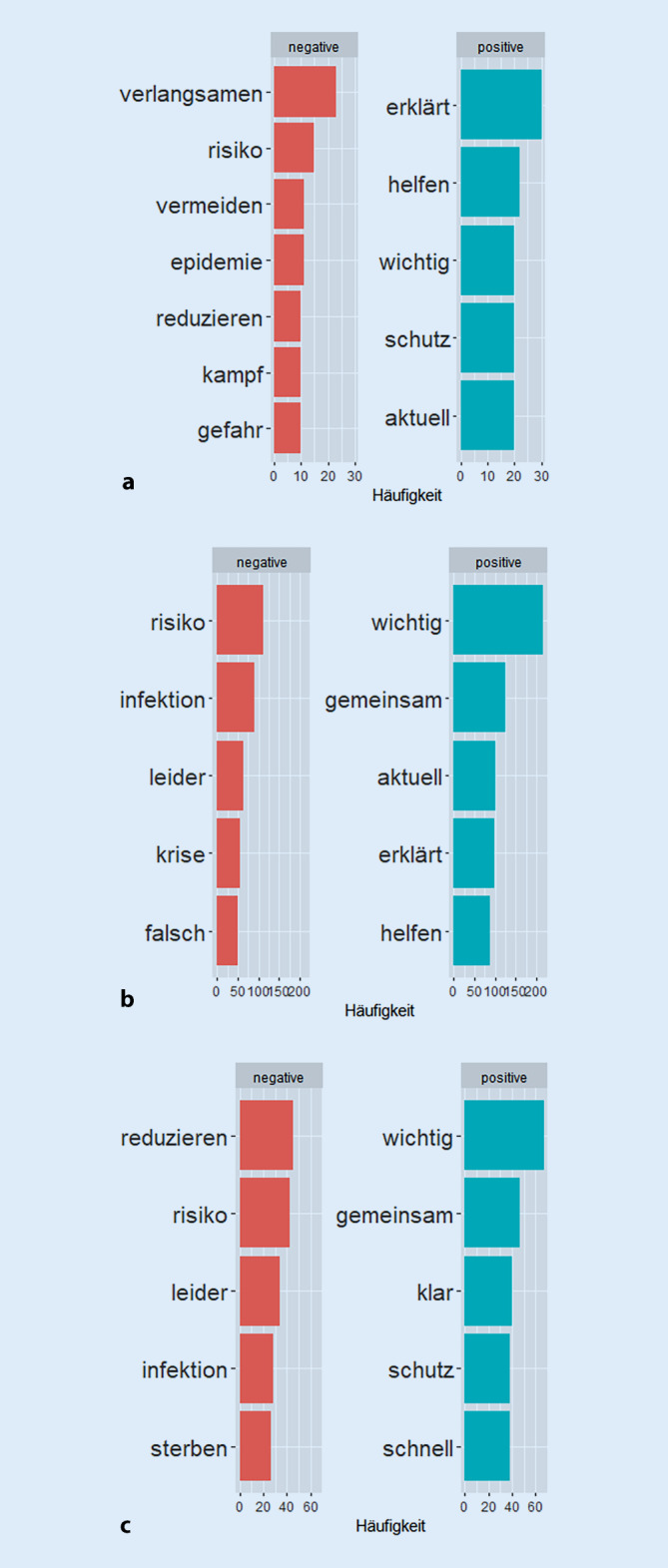

Zur weiteren Analyse wurden die COVID‑19-Tweets der Behörden und Expert*innen in den 3 Phasen auf die Häufigkeit positiv und negativ besetzter Wörter hin untersucht. Als Ergebnis dieses Vergleichs zeigt Abb. 2 die jeweiligen Top 5 dieser Wörter.

In Phase 1 kommt das Wort „verlangsamen“ mit einer negativen Polarität am häufigsten im Datensatz vor, gefolgt von den negativen Wörtern „Risiko“, „vermeiden“, „Epidemie“, „reduzieren“, „Kampf“ und „Gefahr“ (die letzten 3 Wörter mit der jeweils gleichen Häufigkeit). Das häufigste positiv besetzte Wort in der ersten Phase ist das Verb „erklärt“, danach kommen „helfen“, „wichtig“, „Schutz“ und „aktuell“.

In Phase 2 sind andere positiv und negativ besetzte Wörter in den Top 5. Das häufigste genutzte Wort mit einer negativen Polarität lautet nun „Risiko“. Das Wort „Infektion“ ist neu in den Top 5 der Wörter mit negativer Konnotation, gefolgt von „leider“, „Krise“ und „falsch“. Die 5 häufigsten positiv besetzten Wörter sind „wichtig“, „gemeinsam“, „aktuell“, „erklärt“ und „helfen“.

Phase 3 erfasst die zweite Welle der COVID‑19-Pandemie in Deutschland. Die 5 häufigsten Wörter mit negativer Polarität in dieser Phase sind „reduzieren“, „Risiko“, „leider“, „Infektion“ und „sterben“. Das Wort „sterben“ erscheint erstmals in den Top 5. Das Wort „Risiko“ ist das einzige negativ konnotierte Wort, das in allen 3 Phasen in den Top 5 erscheint. In Phase 3 sind die 5 häufigsten positiv besetzten Wörter „wichtig“, „gemeinsam“, „klar“, „Schutz“ und „schnell“. Das Wort „wichtig“ erscheint in allen 3 Phasen in den Top 5.

Abb. 2 verdeutlicht, dass die positiv besetzten Wörter in den untersuchten COVID‑19-Tweets häufiger vorkommen als die negativ besetzten. Die Tatsache, dass die Polarität der Sentiments im Durchschnitt dennoch negativ ist, ist darauf zurückzuführen, dass die negativen Wörter eine stärkere Polarität aufweisen.

**Figure 3:**
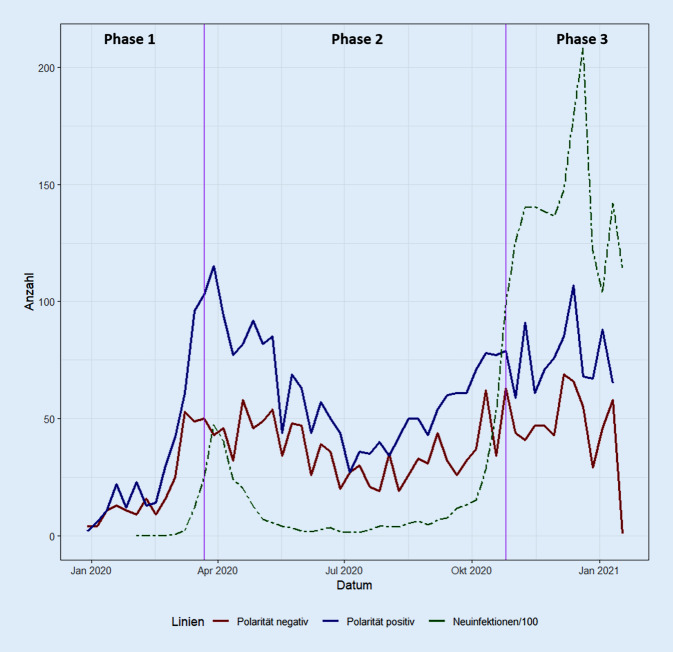

Abb. 3 zeigt die Anzahl der positiv (blau) und negativ (rot) konnotierten COVID‑19-Tweets von Behörden und Expert*innen aggregiert pro Woche (*n* = 8251). Die gepunktete grüne Linie stellt die Anzahl der SARS-CoV-2-Neuinfektionen dar (pro Tag am Stichtag). Die Skala der Neuinfektionen wurde zur besseren Vergleichbarkeit transformiert (/100). In der Darstellung wird deutlich, dass die Sentiments bzw. Emotionen besonders zu Beginn der Pandemie stark ausgeprägt waren. Zwar überwiegen von der Anzahl her die positiven Tweets, aber die negativen sind stärker bezüglich der Polaritätswerte und somit emotionaler als die positiven Tweets mit schwächeren Polaritätswerten. Insgesamt ist der Mittelwert der Tweets deshalb negativ (siehe oben). Die Anzahl der Tweets folgt auch nach Bewertung der Sentiments und Aufteilung in positiv und negativ besetzte Tweets in etwa der Entwicklung der Neuinfektionszahlen in Deutschland. Es zeichnet sich eine erste Welle im März 2020 und der Beginn der zweiten Welle ab Herbst 2020 ab. Auch das Abflauen der Fallzahlen spiegelt sich in deutlich weniger emotionalen Tweets und einer niedrigeren Anzahl an Tweets im Juli und August 2020 wider.

## Diskussion

Die Auswertung zeigt deutlich eine Parallelität zwischen der Emotionalität der COVID‑19-Tweets bei den untersuchten Akteur*innen und der Entwicklung der Neuinfektionen in Deutschland. Auch das Abflauen der Infektionszahlen über den Sommer 2020 zeigt sich in den Emotionen der Wörter der Nachrichten auf Twitter.

Bei der Betrachtung der Sentiments wird erkennbar, dass mehr positiv als negativ besetzte COVID‑19-Tweets veröffentlicht wurden. Allerdings sind die negativ besetzten stärker bezüglich der Polaritätswerte und somit emotionaler als die positiven mit schwächeren Polaritätswerten. Die Sentiments der Krisenkommunikation zur COVID‑19-Pandemie in Deutschland weisen im Untersuchungszeitraum somit eine durchschnittlich negative Polarität auf. Das bedeutet, dass die Krisenkommunikation sowohl der Behörden- als auch der Expert*innen-Tweets gemessen anhand der Sentiments im Durchschnitt negativ konnotiert war. Auffällig ist, dass die Behörden-Tweets nahe der Nulllinie und damit eher im Bereich der Neutralität angesiedelt sind, während die Tweets der Vergleichsgruppe deutlich negativer formuliert sind. Das Abflachen der Emotionen nahe der Neutralität in Phase 2 ist auffällig. Bezogen auf das Ziel dieser Arbeit, ergibt sich hier ein Hinweis für Optimierungspotential der behördlichen Krisenkommunikation in Deutschland. Es sollte für die Zukunft kritisch hinterfragt werden, inwieweit dieser Zeitraum für eine emotionalere Krisenkommunikation seitens der Behörden hätte genutzt werden können, um z. B. auf eine mögliche zweite COVID‑19-Welle vorzubereiten.

Die Sentiments der unabhängigen Expert*innen sind zu Beginn der Pandemie am negativsten und grundsätzlich negativer als die der Behörden. Die Behörden-Tweets sind in Phase 2 am wenigsten emotional. In Phase 3 bekommen deren Tweets wieder eine negativere Konnotation, was mit zunehmender Infektionsaktivität zu erklären ist. Für die Optimierung von Krisenkommunikation sollten Behördenverantwortliche überprüfen, inwieweit die Organisationen zukünftig auch Zeiten mit geringer Infektionsaktivität mit einer eindringlichen Krisenkommunikation nutzen sollten – wie Expert*innen es durchgängiger tun. Schließlich sollte über eine weniger allgemeine und in Teilen sogar emotionalere Wortwahl (mit starken Polaritäten) seitens der Behörden nachgedacht werden, wobei vorher genau zu eruieren wäre, wie stark die Emotionen sein dürfen, ohne dass Panik entsteht oder die Professionalität/Seriosität der Behörde infrage gestellt wird. Hier wäre ein Ansatzpunkt für zukünftige Forschung, um verschiedene Emotionen in der Behördenkommunikation und deren Auswirkungen abzuschätzen.

Die hier untersuchten Häufigkeiten einzelner positiv und negativ besetzter Wörter in den betrachteten 3 Phasen (Abb. 2) spiegeln auch Veränderung in der Krisenkommunikation wider, die die Akteure vorgenommen haben. Die häufigsten negativ konnotierten Wörter in der ersten Phase (z. B. „verlangsamen“, „Risiko“, „vermeiden“, „Kampf“ und „Gefahr“) verdeutlichen die Bemühungen der Akteur*innen um Aufklärung sowie eine Einordnung des Geschehens verknüpft mit Warnungen. Die häufigsten negativ besetzten Wörter der Phase 2 (z. B. „leider“, „Krise“, „falsch“ und „wichtig“, „gemeinsam“, „erklärt“) zeigen, dass schon eine gewisse Routine und Erfahrung mit der Pandemiesituation eingekehrt ist und es nun um weitere Einordnungen geht. Auch der Begriff „Krise“ erscheint bezeichnend, da er die Auswirkungen der Pandemie auf gesundheitlicher, sozialer und politischer Ebene bedeuten kann. Besonders das Auftauchen des Wortes „sterben“ in den Top 5 in der dritten Phase lässt sich ggf. durch die zunehmende Erfahrung mit den Auswirkungen der Pandemie erklären und der bis zu dem Zeitpunkt registrierten Todeszahlen im Zusammenhang mit COVID‑19.

Ziel der Studie war es, wichtige Erkenntnisse zur Optimierung der behördlichen Krisenkommunikation für künftige Ereignisse in Deutschland zu gewinnen. Diese Erkenntnisse sollen die Ergebnisse der vorangegangenen quantitativen Inhaltsanalyse [17] vervollständigen, um die Frage zu beantworten, wodurch der größere Erfolg der unabhängigen Expert*innen bei der Verbreitung von Informationen zu COVID‑19 begründet ist. Die Analyse der Sentiments liefert hier eine weitere Teilantwort. Im Vergleich mit den Behörden wird deutlich, dass externe Expert*innen negativer kommunizierten. Es kann vermutet werden, dass diese Krisenkommunikation der unabhängigen Expert*innen nachhaltiger bei den Rezipienten ankommt als die neutralere Krisenkommunikation seitens der Behörden – und einen Teil des Erfolgs darstellt.

Eine Übersicht über die Entwicklung globaler Sentiments in der COVID‑19-Pandemie liefern Lwin et al. [23]. Im Rahmen einer Inhaltsanalyse englischsprachiger Tweets untersuchten sie die Entwicklung der Emotionen Angst, Wut, Freude und Trauer sowie die Narrative, die diesen zugrunde liegen. Es zeigte sich, dass sich die Sentiments zu COVID‑19 im Untersuchungszeitraum innerhalb weniger Wochen stark verändert haben. Dies ähnelt den hier gefundenen Ergebnissen, wonach sich insbesondere die Sentiments der Behörden im Untersuchungszeitraum deutlich veränderten (Abb. 1). Eine chinesische Studie zeigte, dass Angst in allen COVID‑19-Tweets vorherrschend war [25]. Lwin et al. zeigten ferner, dass während der ersten Phase der Pandemie Angst vorherrschend war, der Anteil ängstlicher Tweets aber fortlaufend abgenommen hat. Die Emotion Wut hat dagegen im Zeitverlauf zugenommen [23]. Diese Differenzierung der verschiedenen Emotionsarten ist mit dem deutschen Sentiment-Lexikon nicht möglich, da dort nur negative, positive und neutrale Sentiments unterschieden werden. Es zeigt sich allerdings, dass die Polarität im Zeitverlauf abnimmt, die Tweets also weniger negativ werden.

Das hier gezeigte Ergebnis der im Durchschnitt negativen Sentiments in COVID‑19-Tweets von Behörden und Expert*innen in Deutschland stimmt mit anderen Studien überein, die auf einem englischsprachigen Textkorpus von Tweets basieren [21, 26]. In diesen Studien zeigte sich, dass auch positive Emotionen zum Ausdruck kamen, wenn es um die Zustimmung zu den Maßnahmen oder Freude über eine gute Gesundheit ging [22–24, 46]. Abb. 2 verdeutlicht, dass in unserer Analyse die Wörter mit positiver Konnotation in den untersuchten COVID‑19-Tweets mit größerer Häufigkeit vorkommen als jene mit negativer. Die Tatsache, dass die Polarität der Sentiments im Durchschnitt negativ ist, liegt aber, wie bereits geschrieben, daran, dass die negativ besetzten Wörter eine stärkere Polarität aufweisen.

Diese Studie weist einige Limitationen auf, wie die Vorgabe des Studiendesigns und die Kategorisierung von Behörden und Expert*innen. Es ist sinnvoll, in zukünftigen Studien über eine weitergehende Definition und Kategorisierung nachzudenken und beispielsweise eine Unterscheidung zwischen Behörden mit hoheitlichen Aufgaben und Wissenschaftsinstitutionen vorzunehmen. Weiterhin muss festgehalten werden, dass ein Vergleich der beiden untersuchten Gruppen, Behörden und unabhängigen Expert*innen, nur eingeschränkt möglich ist, da sich die Möglichkeiten der Kommunikationswege in vielerlei Hinsicht unterscheiden. So stehen Behörden neben den sozialen Medien verschiedenste Kommunikationswege zur Verfügung, wie beispielsweise die Pressekonferenzen des RKI. Auch muss bedacht werden, dass nicht alle Behörden ihre Kommunikationsstrategie auf die Bevölkerung als solche ausgerichtet haben, sondern dass sie Twitter als wichtiges Kommunikations- und Nachrichtenmedium nutzen, um ihre Botschaft über Journalist*innen als Multiplikator*innen zu vermitteln und mit der Fachöffentlichkeit zu kommunizieren. Es bleibt festzuhalten, dass Behörden per se anders kommunizieren müssen als eine Einzelperson dies kann.

Des Weiteren besteht ein Bedarf an der Durchführung ähnlicher Studien, die sowohl mit dem Lexikonansatz als auch mit einer anderen Methode durchgeführt werden. Schließlich ist die Auswahl der Behörden und Expert*innen-Accounts als Limitation anzusehen, die größtenteils extern vorgegeben war. Zukünftig sollte die Auswahl der einzuschließenden Accounts optimiert werden. Vorstellbar ist ein zweistufiges Verfahren. In einem ersten Schritt könnten solche Accounts ausgewählt werden, die viel zu einem Thema twittern. In einem zweiten Schritt könnte hieraus eine Stichprobe gezogen werden und schließlich deren gesamtes Textkorpus untersucht werden. Ferner ist zu diskutieren, inwieweit ein Lexikon die Sentiments über Anwendungsgebiete hinweg beschreiben kann. So kann z. B. infrage gestellt werden, ob „verlangsamen“ im Rahmen von COVID‑19-Sentiment-Analysen nicht eher als positiv besetztes Wort gewertet werden müsste.

## Fazit

Dieser Beitrag präsentiert eine Untersuchung der Sentiments in den Tweets zu COVID‑19 im ersten Pandemiejahr in Deutschland und vergleicht dabei die Sentiments zwischen Behörden auf der einen und unabhängigen Expert*innen auf der anderen Seite. Vor dem Hintergrund vorheriger Studien, in denen Expert*innen-Tweets erfolgreicher sind und gemessen an Retweets und Likes eine höhere Reichweite erzielen, leistet diese Studie damit einen ergänzenden Beitrag zum Verständnis des größeren Erfolgs von Expert*innen im Vergleich zu Behörden.

Konkret verfolgte diese Arbeit das Ziel der Analyse der Sentiments und der Feststellung einer positiv, negativ oder neutral konnotierten Kommunikation in den auf Twitter von beiden Akteursgruppen zu COVID‑19 im ersten Jahr der Pandemie (01.01.2020–15.01.2021) veröffentlichten Tweets. Die Analyse hat gezeigt, dass die Polarität der Sentiments in den Tweets beider Akteursgruppen im Durchschnitt negativ ist, was internationale englischsprachige Sentiment-Studien zu COVID‑19-Tweets bestätigen. Es zeigen sich ferner deutliche Unterschiede in den Polaritätswerten der Tweets zwischen Behörden und Expert*innen. Expert*innen twittern nämlich im Untersuchungszeitraum mit deutlich negativer konnotierten Wörtern über COVID‑19 als Behörden. Aus den gewonnenen Ergebnissen ergeben sich neben Ansätzen für zukünftige Forschung auch Hinweise für die Optimierung behördlicher Krisenkommunikation. Wollen Behörden ähnliche Erfolge in der Krisenkommunikation auf Twitter erreichen wie die unabhängigen Expert*innen, sollte bestimmt werden, ob behördliche Krisenkommunikation emotionaler sein muss bzw. wie emotional sie sein darf, ohne unerwünschte Panik zu erzeugen. Außerdem sollten alternative Strategien für die Krisenkommunikation erarbeitet werden, wie z. B. der Aufbau kommunikativer interner Expert*innen in den Behörden selbst. Auch ist eine stärkere Zusammenarbeit mit unabhängigen Expert*innen im Rahmen vorbereitender Risikokommunikation durch Account-Takeover (zeitliche Übernahme des Behördenaccounts) denkbar, um an den Vorteilen von Einzelaccounts zu partizipieren.

## Footnotes

1 Natural Language Processing (NLP), im Deutschen auch „linguistische Datenverarbeitung“ genannt, ist eine Unterkategorie von künstlicher Intelligenz und bezeichnet die algorithmische Verarbeitung von natürlicher Sprache.

2 Zum Teil kommunizieren diese Expert*innen als „Lab“ (Arbeitsgruppe). Sie sind dennoch von den Behörden zu unterscheiden.

3 Die retrospektive Phaseneinteilung der COVID‑19-Pandemie in Deutschland durch das Robert Koch-Institut (RKI; [33]) wurde erst am 15.04.2021 veröffentlicht und fällt mit dem Projektende der zugrunde liegenden Gesamtstudie zusammen. Daher wurde in dieser Studie mit einer anderen Phaseneinteilung gearbeitet, die sich an den zentralen Ereignissen während der Pandemie orientiert. Das RKI hat bei seiner Einteilung zusätzlich zu den Ferien und den bundespolitischen Maßnahmen auch die Labortestungen, die Meldungen gemäß Infektionsschutzgesetz, die syndromische Surveillance und das Intensivregister herangezogen. Unsere Phase 1 entspricht somit der Phase 0 des RKI, unsere Phase 2 den Phasen 2–3 des RKI und die Phase 3 der Phase 3 des RKI. Wir beziehen uns hier auf die Aktualisierung der retrospektiven Phaseneinteilung durch das RKI [34].

4 Die Namen der Expert*innen sind hier nicht genannt, da der Fokus auf einer Analyse der Gruppe liegt und nicht auf einer individuellen Betrachtung.

5 Auch Negationen wurden untersucht. Dies sind Wörter, die die Bedeutung einer Aussage verneinen. Dazu gehören Wörter wie „kein“ oder „nicht“, die das ursprüngliche Sentiment umkehren (siehe [45]). Aus „Risiko“ wird in Verbindung mit der Negation „kein“ insgesamt ein positiver Ausdruck. Die Überprüfung des Datensatzes auf Negationen und die Berechnung der Sentiments ohne die Negationen führten zu keinem wesentlich anderen Ergebnis als der Einschluss der Negationen: Der Mittelwert der Sentiments der Behörden-Tweets lag bei −0,007 und der Expert*innen bei −0,080.

## References

[1] König M, König W, Klenk T, Nullmeier F, Wewer G (2020). Soziale Medien (Social Media). Handbuch Digitalisierung in Staat und Verwaltung.

[2] Nuernbergk C, Stegbauer C, Clemens I (2020). Das Virus in den sozialen Netzwerken: Corona-Dynamiken am Beispiel politisch-medialer Netzwerke. Corona-Netzwerke – Gesellschaft im Zeichen des Virus.

[3] Ruhrmann G, Daube D, Lohse AW (2021). Die Rolle der Medien in der COVID-19-Pandemie. Infektionen und Gesellschaft.

[4] Ahmed W, Vidal-Alaball J, Downing J, López Seguí F (2020). COVID-19 and the 5G conspiracy theory: social network analysis of Twitter data. J Med Internet Res.

[5] ARD, ZDF (2020) ARD/ZDF-Onlinestudie 2020. Anteil der Nutzer von Social-Media-Plattformen nach Altersgruppen in Deutschland im Jahr 2020. ard-zdf-onlinestudie.de. Zugegriffen: 11. Dezember 2020

[6] Hölig S (2018). Eine meinungsstarke Minderheit als Stimmungsbarometer?! Über die Persönlichkeitseigenschaften aktiver Twitterer. M&K.

[7] Mellon J, Prosser C (2017). Twitter and Facebook are not representative of the general population: political attitudes and demographics of British social media users. Res Polit.

[8] Shahi GK, Clausen S, Stieglitz S (2021) Who shapes crisis communication on Twitter? An analysis of influential German-language accounts during the COVID‑19 pandemic. http://arxiv.org/pdf/2109.05492v1. Zugegriffen: 02.02.2023

[9] Prochazka F, Schützeneder J, Graßl M (2022). Vertrauen in Journalismus unter Social-Media-Bedingungen. Journalismus und Instagram.

[10] Bogner A, Littig B, Menz W, Bogner A, Littig B, Menz W (2014). Wer ist ein Experte? Wissenssoziologische Grundlagen des Expertinneninterviews. Interviews mit Experten.

[11] Acar A, Muraki Y (2011). Twitter for crisis communication: lessons learned from Japan’s tsunami disaster. IJWBC.

[12] Tsubokura M, Onoue Y, Torii HA (2018). Twitter use in scientific communication revealed by visualization of information spreading by influencers within half a year after the Fukushima Daiichi nuclear power plant accident. PLoS ONE.

[13] Cho SE, Jung K, Park HW (2013). Social media use during Japan’s 2011 earthquake: how Twitter transforms the locus of crisis communication. Media Int Aust.

[14] Kostkova P, Szomszor M, Louis SC (2014). #swineflu: the use of Twitter as an early warning and risk communication tool in the 2009 swine flu pandemic. ACM Trans Manage Inf Syst.

[15] Tsao S-F, Chen H, Tisseverasinghe T, Yang Y, Li L, Butt ZA (2021). What social media told us in the time of COVID-19: a scoping review. Lancet Digit Health.

[16] Lyu JC, Luli GK (2021). Understanding the public discussion about the centers for disease control and prevention during the COVID-19 pandemic using Twitter data: text mining analysis study. J Med Internet Res.

[17] Drescher LS, Roosen J, Aue K, Dressel K, Schär W, Götz A (2021). The spread of COVID-19 crisis communication by German public authorities and experts on twitter: quantitative content analysis. JMIR Public Health Surveill.

[18] Kamiński M, Szymańska C, Nowak JK (2020). Whose tweets on COVID-19 gain the most attention: celebrities, political, or scientific authorities?. Cyberpsychol Behav Soc Netw.

[19] de Caro W (2020). Infodemia and COVID-19: a text mining analysis. Eur J Public Health.

[20] Das S, Dutta A (2021). Characterizing public emotions and sentiments in COVID-19 environment: a case study of India. J Hum Behav Soc Environ.

[21] Boon-Itt S, Skunkan Y (2020). Public perception of the COVID-19 pandemic on Twitter: sentiment analysis and topic modeling study. JMIR Public Health Surveill.

[22] Hung M, Lauren E, Hon ES (2020). Social network analysis of COVID-19 sentiments: application of artificial intelligence. J Med Internet Res.

[23] Lwin MO, Lu J, Sheldenkar A (2020). Global sentiments surrounding the COVID-19 pandemic on Twitter: analysis of twitter trends. JMIR Public Health Surveill.

[24] Saleh SN, Lehmann CU, McDonald SA, Basit MA, Medford RJ (2020). Understanding public perception of coronavirus disease 2019 (COVID-19) social distancing on Twitter. Infect Control Hosp Epidemiol.

[25] Xue J, Chen J, Chen C, Zheng C, Li S, Zhu T (2020). Public discourse and sentiment during the COVID 19 pandemic: using Latent Dirichlet Allocation for topic modeling on Twitter. PLoS ONE.

[26] Samuel J, Ali GGMN, Rahman MM, Esawi E, Samuel Y (2020). COVID-19 public sentiment insights and machine learning for tweets classification. Information.

[27] Liu C, Fang F, Lin X (2021). Improving sentiment analysis accuracy with emoji embedding. J Saf Sci Resil.

[28] Shim J-G, Ryu K-H, Lee SH, Cho E-A, Lee YJ, Ahn JH (2021). Text mining approaches to analyze public sentiment changes regarding COVID-19 vaccines on social media in korea. Int J Environ Res Public Health.

[29] Marcec R, Likic R (2021). Using Twitter for sentiment analysis towards AstraZeneca/Oxford, Pfizer/BioNTech and Moderna COVID-19 vaccines. Postgrad Med J.

[30] Hoffmann CP, Högg R, Holenstein M (2022). Eine vergleichende Evaluation der Online-Krisenkommunikation von Behörden und unabhängigen Expert*innen im Zuge der Covid-19 Pandemie als Grundlage für die Verbesserung der BfS-Krisenkommunikation. Vorhaben 3620S72215 und 3620S72216.

[31] ADM Arbeitskreis Deutscher Markt- und Sozialforschungsinstitute e. V., Arbeitsgemeinschaft Sozialwissenschaftlicher Institute e. V., BVM Berufsverband Deutscher Markt- und Sozialforscher e. V., Deutsche Gesellschaft für Online Forschung e. V. (2014) Richtlinie für Untersuchungen in den und mittels der Sozialen Medien (Soziale Medien Richtlinie)

[32] Twitter (2020) Twitter Allgemeine Geschäftsbedingungen. https://twitter.com/de/tos. Zugegriffen: 14. Dez. 2020

[33] Schilling J, Buda S, Fischer M (2021). Retrospektive Phaseneinteilung der COVID-19-Pandemie in Deutschland bis Februar 2021.

[34] Schilling J, Buda S, Tolksdorf K (2022). Zweite Aktualisierung der „Retrospektiven Phaseneinteilung der COVID-19-Pandemie in Deutschland“.

[35] Stieglitz S, Dang-Xuan L, Bruns A, Neuberger C (2014). Social media analytics. Bus Inf Syst Eng.

[36] Zeng D, Chen H, Lusch R, Li S-H (2010). Social media analytics and intelligence. IEEE Intell Syst.

[37] Alamoodi AH, Zaidan BB, Zaidan AA (2020). Sentiment analysis and its applications in fighting COVID-19 and infectious diseases: a systematic review. Expert Syst Appl.

[38] Valle-Cruz D, Fernandez-Cortez V, López-Chau A, Sandoval-Almazán R (2021). Does Twitter affect stock market decisions? Financial sentiment analysis during pandemics: a comparative study of the H1N1 and the COVID-19 periods. Cognit Comput.

[39] Liu B, Indurkhya N (2012). Sentiment analysis and subjectivty. Handbook of natural language processing.

[40] Ortigosa A, Martín JM, Carro RM (2014). Sentiment analysis in Facebook and its application to e-learning. Comput Human Behav.

[41] Mohammad SM, Turney PD (2013) Crowdsourcing a word-emotion association lexicon. Comput Intell 29(3):436–465

[42] Remus R, Quasthoff U, Heyer G (2010). SentiWS—a publicly available German-language resource for sentiment analysis.

[43] Rauh C (2018). Validating a sentiment dictionary for German political language—a workbench note. J Inf Technol Polit.

[44] Thelwall M, Mahrt M, Bruns A, Puschmann C, Burgess J, Weller K (2013). Sentiment analysis and time series with twitter. Twitter and society.

[45] Silge J, Robinson D (2021). Welcome to text mining with R | text mining with R.

[46] Bögenhold D, Marschall J, Stegbauer C (2010). Metapher, Methode, Theorie. Netzwerkforschung in der Wirtschaftssoziologie. Netzwerkanalyse und Netzwerktheorie. Ein neues Paradigma in den Sozialwissenschaften.

[47] Robert Koch-Institut (2021) Täglich gemeldete Neuinfektionen und Todesfälle mit dem Coronavirus (COVID‑19) in Deutschland seit Januar 2020. Abgerufen über Statista. https://de.statista.com/statistik/daten/studie/1100739/umfrage/entwicklung-der-taeglichen-fallzahl-des-coronavirus-in-deutschland/?locale=de. Zugegriffen: 10. Febr. 2021

